# A rare case of uterine leiomyosarcoma: a case report

**DOI:** 10.1186/1752-1947-4-222

**Published:** 2010-07-22

**Authors:** Venkata Sujatha Vellanki, Meghana Rao, Chinna Babu Sunkavalli, Rao N Chinamotu, Shailaja Kaja

**Affiliations:** 1 Department of Obstetrics and Gynaecology, Kamineni Institute of Medical Sciences Sreepuram, Narketpally, Nalgonda - 508254, Andhra Pradesh, India

## Abstract

**Introduction:**

Malignant change in a leiomyoma or uterine fibroid is termed *leiomyosarcoma*. It arises from smooth muscle of the uterus and is a rare tumor that accounts for 2% to 5% of all uterine malignancies. Very few cases are reported in the literature. Our patient did not have any history of genital bleeding, which is the usual presentation in uterine sarcoma. We report an original case report of an unusual presentation of this rare tumor arising from the uterus.

**Case presentation:**

A 40-year-old nulliparous woman of South Indian origin presented with a mass in her abdomen for one year with a rapid increase in size over the previous three months. Tumor marker CA-125 was raised, and a computed tomography scan showed a mass arising from the pelvis. An exploratory laparotomy was performed and the histopathology report confirmed the diagnosis of uterine leiomyosarcoma.

**Conclusion:**

Because of their rarity, uterine sarcomas are not suitable for screening. Diagnosis is by histopathologic examination and surgery is the only treatment. The prognosis for women with uterine sarcoma primarily depends on the extent of disease at the time of diagnosis and the mitotic index.

## Introduction

Malignant change in a leiomyoma or uterine fibroid is termed *leiomyosarcoma*. It arises from smooth muscle of the uterus and is a rare tumor that accounts for 2% to 5% of all uterine malignancies [1].

## Case report

A 40-year-old nulliparous woman of South Indian origin reported to our outpatient clinic with complaints of a mass in the lower abdomen for one year and lower abdominal pain for three months. The patient was apparently asymptomatic one year previously, and then she noticed a mass in the lower abdomen that gradually increased in size. She provided a history of a rapid increase in size for the past three months. She also had associated lower abdominal pain, which was dull and aching in type, dragging in nature and continuous with no aggravating or relieving factors. Her menstrual cycles were regular and normal. She had no history of genital bleeding.

On examination, pallor was present, and the patient's vital signs were normal. She was thinly built. On abdominal examination, an irregular midline mass arising from the pelvis was present. The upper and lateral borders of the mass could be made out; the lower margin could not be ascertained. The mass was firm to hard in consistency with restricted mobility and nontender with no free fluid. There was no hepatosplenomegaly.

On vaginal examination, the patient's uterus was enlarged to 20 weeks' gestational size and nodular, occupying the whole pelvis. No mass could be appreciated separate from uterus. Computed tomography (CT) scan findings were suggestive of a large, multiloculated, multiseptate growth containing both solid and cystic elements arising from the pelvis and extending into the abdomen, measuring 25 × 17 cm, adherent to both the uterus and bladder. CA125 was 94.80 ng/mL (0-35 ng/mL). Other results were normal, and the patient was posted for exploratory laparotomy.

**Figure 1:**
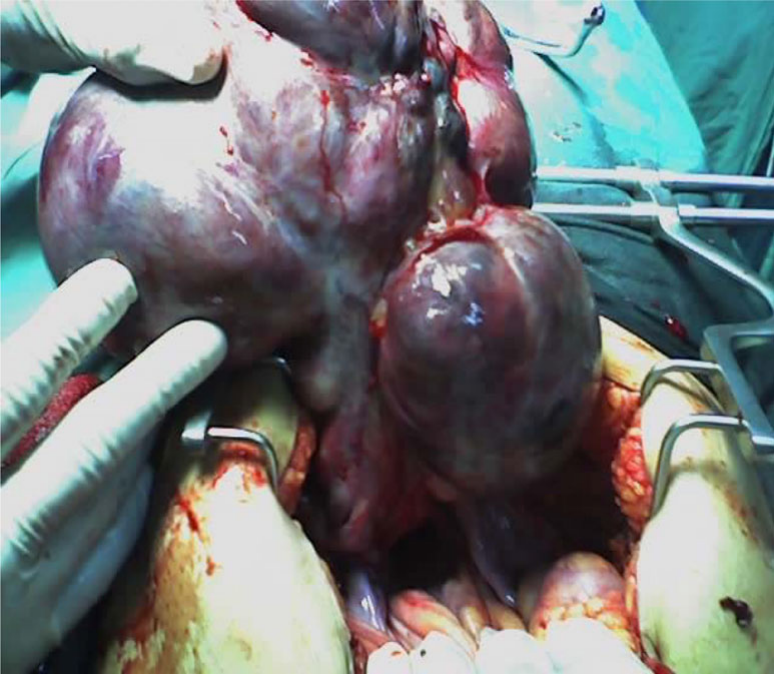
**The uterus with a pedunculated tumor**.

**Figure 2:**
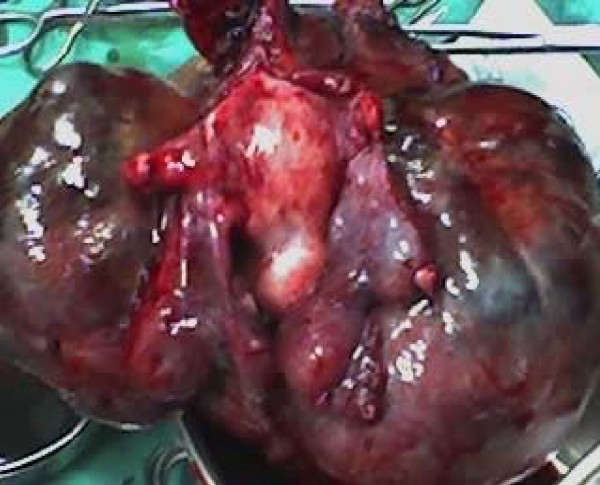
**A lobulated mass arising from the uterine fundus**.

**Figure 3:**
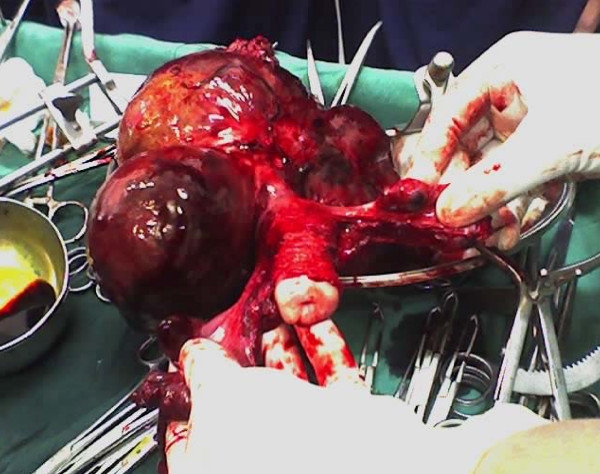
**The uterus, cervix, bilateral fallopian tubes and ovaries and mass from fundus**.

Intraoperatively, omental adhesions to the mass were noticed. A mass of 20 weeks' size was arising from the fundus of the uterus (Figure 1). It was lobulated (Figure 2) with solid, cystic and hemorrhagic components. Both ovaries were normal, and the fallopian tubes were edematous (Figure 3).

**Figure 4:**
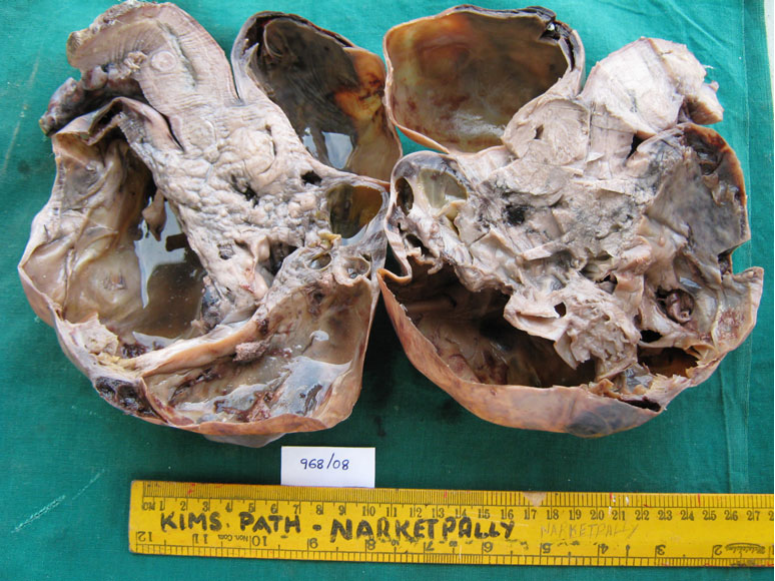
**Cut sections of the uterus and the tumor**.

**Figure 5:**
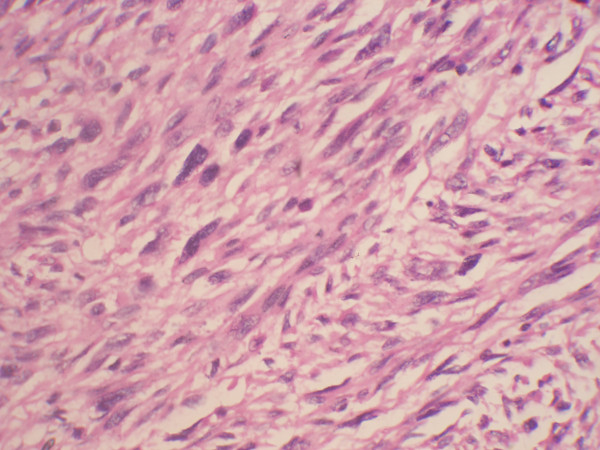
**Microscopic examination showing a cellular tumor arranged in interlacing bundles of spindle cells with elongated hyperchromatic nuclei and nuclear pleomorphism**.

Total abdominal hysterectomy (along with tumor) with bilateral salpingo oophorectomy was done (Figure 4). The uterus measured 8 cm × 5 cm × 3 cm with a subserosal bosselated growth from the fundus measuring 18 cm × 15 cm × 11 cm with variable consistency. Omental biopsy and external iliac lymph node biopsy were taken and sent for histopathologic examination. Histopathologic examination showed a cellular tumor arranged in interlacing bundles of spindle cells with elongated hyperchromatic nuclei. The tumor cells were exhibiting moderate pleomorphism and bizarre nuclei with multinucleate tumor giant cells. There were scattered areas and normal and abnormal mitotic figures (> 4/high-power field) with marked nuclear atypia suggestive of uterine leiomyosarcoma (Figure 5). The omentum and external iliac lymph node were free from tumor cells.

## Discussion

Uterine sarcomas are rare and constitute about 2% to 5% of all uterine malignancies [1]. Prior pelvic radiation (10%-25% of cases) was considered to be a risk factor. Data regarding parity and time of menarche and menopause as risk factors are inconclusive. An increase in the risk of uterine sarcomas appears to accompany the use of long-term adjuvant tamoxifen in women with breast cancer [2]. The incidence of sarcoma is 1% to 2% in postmenopausal women. These patients usually present with abnormal uterine bleeding [3]. Our patient did not have any menstrual abnormalities. It is a rapidly growing tumor with a doubling time of four weeks. The incidence of mixed Müllerian sarcomas is 50% percent, leiomyosarcoma is 30%, endometrial stromal sarcoma is 15%, and adenosarcoma is 5%. The diagnosis of uterine sarcomas is made from histologic examination of the entire uterus.

## Conclusion

Because of their rarity, uterine sarcomas are not suitable for screening. Surgery is the only treatment. The prognosis for women with uterine sarcoma primarily depends on the extent of disease at the time of diagnosis and the mitotic index [4].Women with tumor size more than 5 cm in maximum diameter have a poor prognosis [5].Nonrandomized studies have reported improved survival after adjuvant chemotherapy with or without radiation therapy. The value of pelvic radiation therapy has not been established. Current studies consist primarily of phase II chemotherapy trials for patients with advanced disease [6].

## Consent

Written informed consent was obtained from the patient for publication of this case report. A copy of the written consent is available for review by the Editor-in-Chief of this journal.

## Competing interests

The authors declare that they have no competing interests.

## Authors' contributions

The case was managed by CNR, VVS, MR, KS and SCB. All authors have read and approved the final manuscript.
